# Assessment of Disease Burden Among Japanese Patients With Pulmonary Sarcoidosis: A Questionnaire-Based Study

**DOI:** 10.7759/cureus.94425

**Published:** 2025-10-12

**Authors:** Hiromi Tomioka, Hiroaki Sato, Mutsumi Kanakubo, Shotaro Maeda, Maki Yokota

**Affiliations:** 1 Respiratory Medicine, Kobe City Medical Center West Hospital, Kobe, JPN; 2 Patients' Association, Sarcoidosis Tomonokai, Osaka, JPN; 3 Medical Affairs, Kyorin Pharmaceutical Co. Ltd., Tokyo, JPN

**Keywords:** fatigue assessment scale, health-related quality of life, king’s sarcoidosis questionnaire, pulmonary sarcoidosis, questionnaire study, small fiber neuropathy screening list

## Abstract

Introduction: Sarcoidosis is a systemic granulomatous disease that can form lesions in almost all organs and most commonly affects the lung. It often causes systemic symptoms such as generalized pain and fatigue, leading to reduced health-related quality of life (HRQoL). Although oral corticosteroids (OCS) are commonly used to manage sarcoidosis, they are associated with side effects and reduced HRQoL. In Japan, data on patient-reported outcomes remain limited.

Methods: This study assessed the disease burden in 158 Japanese patients with pulmonary sarcoidosis, recruited through a sarcoidosis patients’ association, using a mailed questionnaire that included three patient-reported outcome measures: the King’s Sarcoidosis Questionnaire (KSQ), Fatigue Assessment Scale (FAS), and Small Fiber Neuropathy Screening List (SFNSL).

Results: The mean age of patients was 68.4 years, and 85.4% were women. The median KSQ General Health Status score was 54.3, indicating a reduced HRQoL. Fatigue was prevalent, with 72.3% of patients scoring ≥22 on the FAS. Small fiber neuropathy (SFN) was suspected in 79.7% of patients with SFNSL scores ≥11. Patients receiving higher doses of OCS (n = 40) had significantly lower KSQ medication scores compared with those receiving lower doses of OCS (median 61.7 vs. 80.9, p = 0.001), suggesting a higher treatment burden. Additionally, OCS users exhibited a higher prevalence of extreme fatigue (FAS ≥35, 26% vs. 14%, p = 0.030) and suspected SFN (SFNSL ≥11, 90% vs. 70%, p < 0.001).

Conclusion: These findings quantitatively demonstrate the burden of systemic symptoms, particularly fatigue and symptoms associated with SFN, in Japanese patients with pulmonary sarcoidosis, with the present study being the first to evaluate KSQ scores in Japan. Even though this study could not establish causality due to its cross-sectional nature, the results underscore the importance of regularly monitoring and managing fatigue and symptoms associated with SFN in patients undergoing treatment with OCS. Such insights may support clinical decision-making and could contribute to optimizing management while minimizing HRQoL impairment.

## Introduction

Sarcoidosis is a systemic granulomatous disease of unknown etiology that primarily affects the lungs, lymph nodes, eyes, and skin and can form lesions in almost all organs and tissues, including the heart, nervous system, bones, muscles, and liver, with the potential of multiple organ involvement [1]. Patients commonly present with organ-specific symptoms such as cough, dyspnea, visual disturbances, skin lesions, arrhythmia, neurological deficits, muscle masses, and bone pain, as well as nonspecific symptoms including generalized pain and fatigue [2,3]. These symptoms may be chronic and refractory to treatment in some patients. The disease has a slightly higher incidence in women, with Japanese reports showing rates per 100,000 population of 0.73 for men and 1.28 for women in 2004 [4]. Pulmonary involvement is the most common organ manifestation, occurring in 86% of Asian patients and 95% of White and Black patients [1,4]. Thus, sarcoidosis shows racial and geographic variations in its clinical manifestations and pathophysiology. Similarly, healthcare systems and access to medical care also differ across countries and regions.

Sarcoidosis has been reported to cause physical, psychological, and social burdens on patients, significantly reducing their quality of life (QOL) [5,6]. In particular, patients frequently experience organ-nonspecific symptoms such as fatigue and symptoms associated with small fiber neuropathy (SFN) [7,8]. A collaborative study from Denmark, Germany, and the Netherlands showed that >80% of patients with sarcoidosis experience fatigue and symptoms associated with SFN and that these symptoms contribute to a significant decrease in QOL [9].

Sarcoidosis is a complex disease, and organ-specific symptoms and laboratory findings do not always reflect systemic symptoms and QOL. However, patients place great importance on QOL improvement as a treatment outcome, and this gap presents a challenge in patient care [10]. Symptoms such as fatigue and pain are often under-evaluated in daily clinical practice. Corticosteroids, commonly used to manage sarcoidosis, can alleviate symptoms but may also cause side effects depending on the dosage and duration, which can further reduce QOL [11,12]. Therefore, a comprehensive approach that considers both treatment burden and patient QOL is required.

In Japan, few studies have assessed the disease burden of sarcoidosis using patient-reported outcome measures (PROMs) [13,14]. This study aimed to evaluate the daily-life burden of Japanese patients with sarcoidosis using PROMs, including the King’s Sarcoidosis Questionnaire (KSQ), Fatigue Assessment Scale (FAS^©^; ild care foundation (www.ildcare.nl)), and Small Fiber Neuropathy Screening List (SFNSL^©^; ild care foundation) [15-17].

## Materials and methods

Data sources and eligible participants

This was a cross-sectional questionnaire-based survey of patients with pulmonary sarcoidosis in Japan, in which three PROMs were used to investigate the patients’ disease burden. The survey was conducted between August and September 2024. Eligible participants were patients aged ≥18 years who were currently suffering from or had suffered from pulmonary sarcoidosis among those who belonged to a sarcoidosis patients’ association, the “Sarcoidosis Tomonokai”, as of July 2024. Pulmonary involvement is the most common organ manifestation of sarcoidosis; hence, this study focused on patients with pulmonary sarcoidosis to capture these characteristics. Participants were recruited from the “Sarcoidosis Tomonokai”, which may limit the generalizability of the findings due to potential recruitment bias, as these members might be more engaged with their disease management or have more severe symptoms than the general population. These findings are expected to deepen healthcare providers’ understanding of the burden on patients with pulmonary sarcoidosis and lead to proposals for more appropriate support. Furthermore, this study is anticipated to contribute to the improvement in the QOL of patients with sarcoidosis.

Recruitment

A semi-quantitative, anonymous, self-administered questionnaire was jointly developed by patients with sarcoidosis, clinicians, and pharmaceutical company representatives to investigate the actual burden of sarcoidosis and its treatment, as well as patients’ expectations regarding medical treatment. The questionnaire was mailed to the members of the patients’ association, together with a consent form. Participants who agreed to participate in the survey returned a signed consent form and questionnaire to the research company (INTAGE Research, Inc.). INTAGE Research compiled the questionnaires collected from the respondents as anonymous data that could not be used to identify individuals and provided them to the research organization (INTAGE Healthcare Inc.). Further, INTAGE Healthcare cleaned the data and provided it to the research group as an analytical dataset. Participants received appropriate compensation for their cooperation in the survey.

Variables

We collected data on patient background (e.g., age, sex), lifestyle (e.g., smoking history), medical history and comorbidities, sarcoidosis treatment (including oral corticosteroids (OCS) use), patient-reported organ involvement (lungs, eyes, skin, heart, etc.), patient-reported outcomes (PROs), and preferences for medical care, including treatment, certification of intractable diseases in Japan, and other factors. The PROMs used in this study included the KSQ [15], the FAS [16], and the SFNSL [17]. The KSQ is a validated, self-administered health-related quality of life (HRQoL) tool specifically designed for patients with sarcoidosis. It comprises five domains - general health status (GHS), lungs, skin, eyes, and medications - each of which generated a score ranging from 0 to 100. Higher scores reflected better health status. The KSQ is widely used in clinical research and practice to assess the multidimensional impact of sarcoidosis and effectively monitor therapeutic outcomes. Furthermore, FAS and SFNSL are validated PRO tools. The FAS was developed to assess the severity of fatigue in patients, including those with sarcoidosis. It consists of 10 items scored on a 5-point Likert scale (total score 10-50), with higher scores indicating greater fatigue; scores ≥22 identify clinically significant fatigue, and scores ≥35 denote extreme fatigue. The SFNSL was designed to screen for the symptoms of SFN. It comprises 21 items scored on a 5-point Likert scale (total score 0-84), with higher scores reflecting more severe SFN symptoms; scores ≥11 indicate probable or highly probable SFN, and scores ≥49 indicate certain SFN.

Statistical analyses

Continuous variables were summarized as mean and standard deviation (SD) or median and interquartile range (IQR), as appropriate. Categorical variables were presented as frequencies and percentages. Fisher's exact test was employed for between-group comparisons of categorical variables. Continuous variables were compared using Student's t-test or Wilcoxon rank-sum test for two groups, and one-way analysis of variance (ANOVA) or Kruskal-Wallis test was used for multiple groups, based on data distribution. Pearson correlation coefficient or Spearman's rank correlation coefficient was calculated to evaluate correlations between variables. To evaluate the differences in FAS and SFNSL between the groups with and without OCS use, the scores were dichotomized at the two thresholds previously described (FAS: ≥22 and ≥35; SFNSL: ≥11 and ≥49). Multivariable logistic regression analyses were performed with adjustments for age, sex, and comorbidities to estimate odds ratios (95% confidence intervals (CI)) and p-values. All statistical tests were two-sided, and the significance level was set at p < 0.05. Missing data were not imputed, and analyses were performed based only on the available data. Analyses were conducted using R 4.4.0 (R Foundation for Statistical Computing, Vienna, Austria) and JMP software package version 17 (SAS Institute Inc., Cary, NC, USA).

Ethics

This study was conducted in compliance with the “Ethical Guidelines for Life Science and Medical Research Involving Human Subjects” (Revised on March 27, 2023) [18], and with the approval of the Research Ethics Review Committee (Approval No. 568, approved on June 18, 2024). The study was registered in the Japan Registry of Clinical Trials (jRCT1030240242). Participation was voluntary, all participants provided written informed consent, and the data were collected and analyzed anonymously. Participants were free to withdraw from the study at any time without providing a reason.

## Results

Enrolled participants and disposition

**Figure 1:**
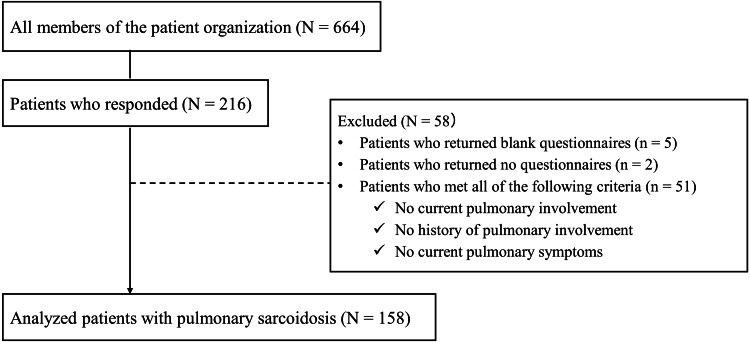
Participants disposition.

Of the 664 members of the patients’ association to whom the questionnaire was sent by postal mail, 216 (32.5%) responded. Of these, 58 patients were excluded, including those who had never had lung lesions. Finally, 158 patients who met the eligibility criteria were analyzed (Figure 1).

Participants’ characteristics

**Figure 2:**
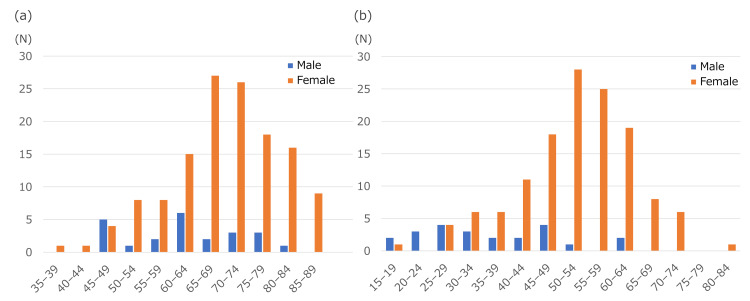
Distribution of current age and age at first diagnosis of sarcoidosis. Distribution of (a) current age in patients with pulmonary sarcoidosis and (b) age at sarcoidosis diagnosis by sex.

The mean age (SD) was 68.4 years (10.8), and the majority comprised women (85.4%). The mean age (SD) at diagnosis was 49.7 years (12.7), and the mean disease duration (SD) was 18.8 years (12.3). The distribution of current age and age at diagnosis according to sex is shown in Figure 2. By occupation at the time of survey, most patients were homemakers (n = 56, 35.4%) and unemployed individuals (n = 55, 34.8%). Participants’ characteristics and details of sarcoidosis lesions are summarized in Tables 1 and Appendix A, respectively.

**Table 1:** Patients’ characteristics.

Variables	Total (N = 158)
Sex, n (%)	
Female	135 (85.4)
Age	
Mean (SD)	68.4 (10.8)
Age category, n (%)	
<40 years	1 (0.6)
40-49 years	10 (6.3)
50-59 years	19 (12)
60-69 years	50 (31.6)
70-79 years	50 (31.6)
≥80 years	26 (16.5)
No response	2 (1.3)
Age at diagnosis of sarcoidosis, years	
Mean (SD)	49.7 (12.7)
Duration of sarcoidosis, years	
Mean (SD)	18.8 (12.3)
Type of employment, n (%)	
Homemaker	56 (35.4)
Unemployed	55 (34.8)
Company employee	14 (8.9)
Part-time worker	12 (7.6)
Self-employed	10 (6.3)
Other	8 (5.1)
Company officer	2 (1.3)
Public officer	1 (0.6)
Payment of specific medical expenses, n (%)	
Yes	102 (64.6)
No	51 (32.3)
No response	5 (3.2)

The most frequently reported comorbidities were osteoporosis (n = 44, 27.8%) and cancer (n = 29, 18.4%). Most participants reported an average sleep duration of five to six hours per night (n = 55, 34.8%). Current smoking was rare (n = 4, 2.5%), and 13.9% (n = 22) reported regular alcohol consumption (Table 2).

**Table 2:** Patient comorbidities and lifestyle factors.

Variables	Total (N = 158)
Comorbidities, n (%)	
Asthma	19 (12)
Diabetes	14 (8.9)
Osteoporosis	44 (27.8)
Depression	8 (5.1)
Rheumatoid	4 (2.5)
Cancer	29 (18.4)
Breast	6 (3.8)
Colon	5 (3.2)
Lung	4 (2.5)
Thyroid	2 (1.3)
Kidney	1 (0.6)
Stomach	1 (0.6)
Skin	1 (0.6)
Uterus	1 (0.6)
Average sleep time, n (%)	
<5 hours	28 (17.7)
5-6 hours	55 (34.8)
6-7 hours	48 (30.4)
7-8 hours	18 (11.4)
8-9 hours	5 (3.2)
≥9 hours	4 (2.5)
Smoking status, n (%)	
Does not smoke	132 (83.5)
Used to smoke but has not smoked for over a month	20 (12.7)
Smokes every day	4 (2.5)
Occasionally smokes	1 (0.6)
No response	1 (0.6)
Drinking habit, n (%)	
Yes	22 (13.9)
No	135 (85.4)
No response	1 (0.6)

Of the 158 patients, 81 (51.3%) received OCS for sarcoidosis, 36 (22.8%) had received them previously, and 39 (24.7%) had never received them. Among current users (n = 74, 47.5%), the mean (SD) daily dose of prednisolone equivalents was 5.3 mg (5.4), with a median (IQR) of 5 mg (2.1) (Table 3).

**Table 3:** Medications for sarcoidosis. ^†^ Of the patients who reported receiving OCS, 74 patients (47.8%) provided dosage information. The dosage is shown as prednisolone equivalents. OCS: oral corticosteroids

Variables	Total (N = 158)	Variables	Total (N = 158)
OCS use, n (%)		Medication other than OCS, n (%)	
Currently	81 (51.3)	Methotrexate	20 (12.7)
Previously	36 (22.8)	Azathioprine	0 (0.0)
Never	39 (24.7)	Leflunomide	0 (0.0)
No response	2 (1.3)	Mycophenolate mofetil	0 (0.0)
OCS dose/day, mg^†^		Infliximab	0 (0.0)
Mean (SD)	5.3 (5.4)	Adalimumab	2 (1.3)
Median (IQR)	5.0 (2.1)	Etanercept	0 (0.0)
OCS dose/day category, n (%)		Nintedanib	0 (0.0)
<5mg	34 (42.0)	Minomycin	3 (1.9)
≥5mg	40 (49.4)	Cardiac pacemaker	23 (14.6)
Unknown	7 (8.6)	Home oxygen therapy	3 (1.9)
-	-	Inhaled corticosteroid	14 (8.9)

Of the 117 patients who were receiving or had received OCS, 58 (49.6%) discontinued OCS use. The primary reason for discontinuation was symptom improvement (n = 48, 41.0%). However, 19 patients (16.2%) discontinued due to concerns about side effects, with eight patients discontinuing on their own judgment and 10 based on a doctor's decision. The proportion of patients who discontinued by their own judgment was relatively high compared with other reasons (Table 4).

**Table 4:** Steroid discontinuation experience and reasons for discontinuation. * Based on 117 participants with steroid use experience. ** Multiple responses allowed.

Steroid discontinuation experience, n (%)*							
Yes	58 (49.6)	Reason for discontinuation, n (%)*^,^**		Decision maker, n (%)*^,^**			
				Physician	Self	Other	No response
		Symptoms improved	48 (41.0)	42 (35.9)	4 (3.4)	1 (0.9)	1 (0.9)
		Side effects occurred	21 (17.9)	19 (16.2)	2 (1.7)	0 (0.0)	0 (0.0)
		No effect observed	17 (14.5)	16 (13.7)	1 (0.9)	0 (0.0)	0 (0.0)
		Concern about side effects	19 (16.2)	10 (8.5)	8 (6.8)	1 (0.9)	0 (0.0)
No	53 (45.3)						
No response	6 (5.1)						

Participants’ HRQoL

**Figure 3:**
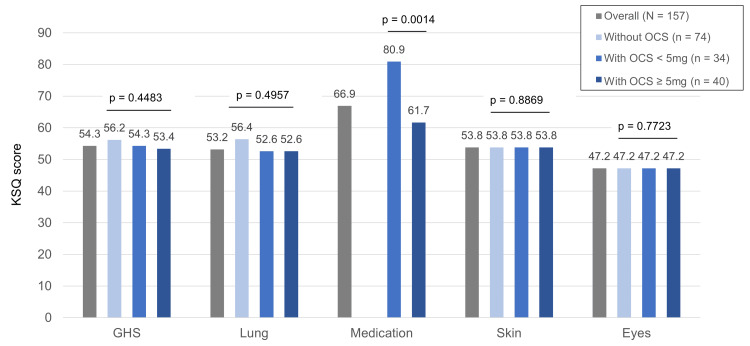
Each domain score on the KSQ stratified by OCS usage. Bar graphs show the median KSQ sub-scores, including GHS, lung, medication, skin, and eyes, for the total population and the following groups: without OCS use, with OCS at <5 mg, and with OCS at ≥5 mg per day. The KSQ lung, skin, and eye scores were calculated only in patients with organ involvement at the respective sites. The KSQ Medication score did not include data for the group without OCS use, as this group was not eligible for score calculation. P-values were calculated using the Kruskal-Wallis test for all sub-scores except for medication, for which the Wilcoxon rank-sum test was used. Effect sizes are reported as eta-squared (≤0 for GHS, lung, skin, eyes) and r = 0.372 for medication. KSQ: King’s Sarcoidosis Questionnaire; GHS: general health status; OCS: oral corticosteroids

For the KSQ GHS, the overall median (IQR) score was 54.3 (45.3-63.9), whereas it was 54.3 (45.3-63.9) in patients with lesions in the lung, 54.3 (45.3-61.9) in the eyes, 54.3 (45.3-63.9) in the skin, and 53.4 (41.7-61.9) in the heart. The median (IQR) KSQ GHS scores by OCS dose per day were 56.2 (43.5-63.9) for patients who did not receive OCS; 54.3 (45.8-63.4), <5 mg OCS; and 53.4 (45.3­-58.0), ≥5 mg OCS (Figure 3). The median (IQR) KSQ Medication scores by OCS dose per day were 80.9 (66.9-100.0) for patients receiving <5 mg and 61.7 (47.0-80.9) for those receiving ≥5 mg, with the latter being significantly lower. The details of each KSQ sub-score analyzed in patients with organ involvement are shown in Appendix B.

**Figure 4:**
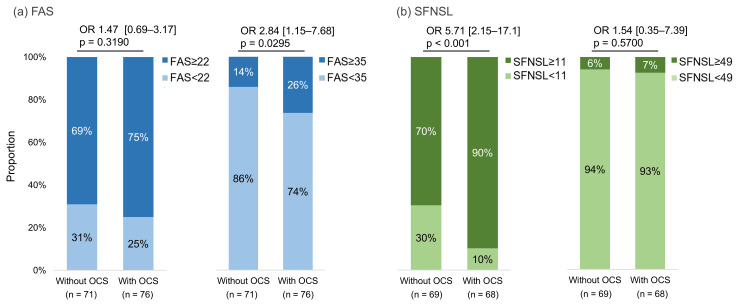
Association between FAS and SFNSL score classification and OCS use. (a) The FAS scores were classified as ≥22 (with fatigue) or <22 and ≥35 (with extreme fatigue) or <35. (b) The SFNSL classification was ≥11 (suspected SFN) or <11 and ≥49 (with SFN) or <49. P-values and odds ratios (ORs) with 95% confidence intervals were derived from logistic regression analysis after adjusting for sex, age, and comorbidities. FAS: Fatigue Assessment Scale; OCS: oral corticosteroids; SFN: small fiber neuropathy; SFNSL: Small Fiber Neuropathy Screening List

The mean FAS score (SD) of the participants was 27.3 (8.6). When stratified by FAS score, 41 participants (27.7%) had a score of <22; 76 (51.4%), 22-34 (fatigue); and 31 (20.9%), ≥35 (extreme fatigue) (Table 5). The higher the FAS severity classification, the higher the proportion of participants who were “currently receiving” OCS and the proportion of participants who held a certificate for payment of specific medical expenses. The logistic regression analysis showed that the proportion of patients with fatigue or extreme fatigue (cut-off score of 22 or 35) in the FAS differed depending on whether they were receiving OCS. There was no significant difference between the two groups when the cut-off score was 22 (p = 0.319). In contrast, when the cut-off score was 35, the proportion of patients with an FAS score of ≥35 was significantly higher in the group receiving OCS (p = 0.030) (Figure 4a).

**Table 5:** Participants’ background by FAS and SFNSL score. FAS: Fatigue Assessment Scale; SFNSL: Small Fiber Neuropathy Screening List; OCS: oral corticosteroids

Variables	FAS (N = 148)			SFNSL (N = 138)		
	<22 point, n = 41 (27.7%)	22-34 point, n = 76 (51.4%)	≥35 point, n = 31 (20.9%)	<11 point, n = 28 (20.3%)	11-48 point, n = 101 (73.2%)	≥49 point, n = 9 (6.5%)
Age, years						
Mean (SD)	66.2 (8.7)	68.8 (11.0)	68.7 (12.3)	63.8 (11.1)	68.3 (10.7)	71.3 (10.4)
Sex, n (%)						
Female	35 (85.4)	64 (84.2)	27 (87.1)	22 (78.6)	86 (85.1)	8 (88.9)
OCS use, n (%)						
Currently	19 (46.3)	37 (48.7)	20 (64.5)	7 (25.0)	56 (55.4)	5 (55.6)
Payment of specific medical expenses, n (%)						
Yes	25 (61.0)	48 (63.2)	22 (71.0)	14 (50.0)	67 (66.3)	8 (88.9)

**Figure 5:**
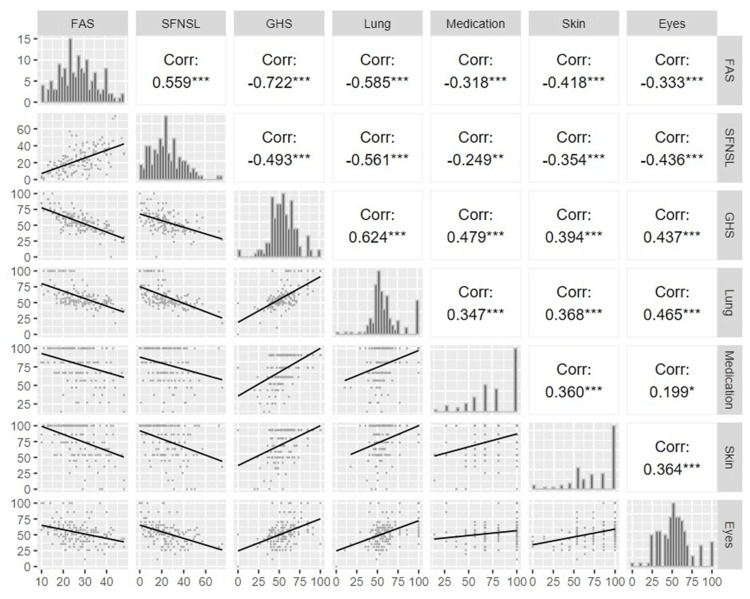
Correlation between KSQ sub-scores and FAS, and SFNSL. Correlations were derived from the Spearman rank correlation test. * <0.05, **<0.01, ***<0.001 The bottom left shows scatter plots with regression lines. The top right presents correlation coefficients among KSQ sub-scores and FAS and SFNSL scores. Between them, the distribution of each score is shown. Corr: correlation; GHS: general health status; KSQ: King’s Sarcoidosis Questionnaire; FAS: Fatigue Assessment Scale; SFNSL: Small Fiber Neuropathy Screening List

For the SFNSL scores, in addition to the total score, a summary of participants’ backgrounds when they were grouped based on the two cut-offs (11 and 49) is shown (Table 5). The mean (SD) overall SFNSL score was 24.0 (14.7), and 28 (20.3%) patients had a score of <11; 101 (73.2%), 11-48; and 9 (6.5%), ≥49. In the logistic regression analysis, when the cut-off was 49, five patients received OCS and four patients did not receive OCS, indicating no statistically significant difference. In contrast, when the cut-off value was 11, the proportion of patients with SFNSL scores of ≥11 was significantly higher in the group receiving OCS (p < 0.001) (Figure 4b). The correlations among the KSQ, FAS, and SFNSL scores are shown in Figure 5. Correlations were observed between the scores of KSQ GHS and FAS, SFNSL, KSQ Lung, and KSQ Medication, as well as between FAS and KSQ Lung, SFNSL, and SFNSL and KSQ Lung. A graphical abstract that overviews the study design and main results is provided in Appendix C.

## Discussion

We quantitatively assessed the multifaceted disease burden in patients with pulmonary sarcoidosis using three PROMs: KSQ, FAS, and SFNSL. Notably, this is the first report to evaluate KSQ scores in Japanese patients. The median (IQR) KSQ-GHS score in this study was 54.3 (45.3-63.9). The median (IQR) FAS score was 27 (21-33), and among the 148 patients whose FAS scores were assessed, 107 (72.3%) had a score of ≥22, indicating the presence of fatigue. The median (IQR) SFNSL score was 22 (13-32), and among the 138 patients whose SFNSL scores were assessed, 110 (79.7%) had a score of ≥11, suggesting suspected SFN. These findings suggest that many Japanese patients with pulmonary sarcoidosis experience organ-nonspecific systemic symptoms such as fatigue and symptoms associated with SFN.

Background

This survey targeted members of a sarcoidosis patients’ association in Japan. Patients belonging to such associations may have more severe symptoms or greater burdens in daily life; therefore, they may not necessarily represent the general population of patients with pulmonary sarcoidosis in Japan. The background characteristics of the study population are described below.

The respondents were mainly in their 60s and 70s and had long-term sarcoidosis, with a median age at diagnosis of 52 years, similar to a previous Japanese epidemiological report [19]. The age distribution at diagnosis showed a bimodal pattern in men and a unimodal peak in their 50s for women, consistent with previous Japanese epidemiological studies [19,20]. Although the number of male patients in this study was relatively small, the overall trend was consistent with that of previous reports. One possible reason for the lower proportion of male patients in this study was the relatively high remission rate of pulmonary sarcoidosis lesions in younger individuals [21]. As a result, many male patients with early-onset pulmonary sarcoidosis may have achieved remission and therefore did not join the sarcoidosis patients’ association.

In this study, 85.4% of respondents were female, compared with 63.5% in a Japanese epidemiological survey of newly diagnosed patients [19]. This proportion aligns with the 84.4% female ratio of patients’ association members, suggesting that the results reflect the characteristics of patients’ association members more strongly than those of the general population of Japanese patients with pulmonary sarcoidosis.

The higher incidence of heart involvement (32.9%) compared to previous epidemiological studies (23.0%) [4] and the high proportion of patients holding medical certificates (64.6%) suggest that patients’ association members tend to represent a cohort with more severe disease than that in the general patient population. Additionally, the higher incidence of eye (male: 26.1%, female: 56.3%) and skin involvement (male: 4.3%, female: 30.4%) among women was consistent with previously reported clinical characteristics of Japanese patients with sarcoidosis [19].

Regarding treatment, 81 patients (51.3%) were currently receiving OCS, with 40 receiving ≥5 mg per day. Generally, patients without cardiac involvement or mild symptoms who are expected to undergo spontaneous remission are monitored without treatment. Studies targeting outpatient populations in Japan have reported treatment rates with OCS ranging from 20 to 35% [14,22], suggesting that the participants in this study represent a cohort with more severe disease.

Other treatments included methotrexate in 20 patients (12.7%) and adalimumab in two patients (1.3%). The high proportion of patients (23 cases, 14.6%) with cardiac pacemakers further supports the presence of severe cases in this study population. However, reports on non-OCS treatments for sarcoidosis in Japan are limited, and the rate of use of such treatments was lower than that in Western countries. One key factor contributing to this difference is that, in Japan, non-steroid treatment for pulmonary sarcoidosis is not covered by health insurance.

Patient-reported outcomes (PROs)

Among the 157 patients in this study who were eligible for KSQ GHS evaluation, their KSQ GHS scores were lower than the median score of 67.9 (IQR: 51.9-80.2) reported in a German cohort study of respiratory department outpatients and the mean KSQ GHS score of 60.6 for the lower quartile of forced vital capacity (FVC) % predicted in a study of mild cases [23]. These findings suggest that the patients in this study tended to have lower HRQoL.

Furthermore, when comparing scores by OCS dosage, the KSQ Medication score was lower in the group receiving ≥5 mg per day compared to that in the group taking <5 mg per day. In this cross-sectional study, we found that patients receiving higher doses of OCS experienced a greater treatment burden. However, due to the cross-sectional design, causality cannot be inferred; the association may reflect confounding by indication, as patients with more severe disease were more likely to receive higher OCS doses.

Regarding the FAS score, the mean (SD) FAS score in this study was 27.3 (8.6); 107 patients (72.3%) met the cutoff value ≥22, which indicates the presence of fatigue. In a previous Japanese study [14], the mean (SD) FAS score was 22.7 (8.2), and 49% of patients had a score ≥22. Compared with that study, our cohort had a higher mean age, longer disease duration, and a greater proportion of patients using OCS (51.3% vs. 20.0%), which may partly explain the higher FAS scores observed, suggesting a greater symptom burden and disease severity in our participants.

The prevalence of fatigue in patients with sarcoidosis ranges from 70% to 83.2% [16,24,25]. Patients undergoing steroid treatment experience more severe fatigue [11].

The mean (SD) SFNSL score was 24.0 (14.7), and 110 patients (79.7%) had SFNSL scores ≥11, indicating suspicion of SFN. In contrast, a previous Japanese study [14] reported a mean (SD) SFNSL score of 13.8 (14.3), with 41.8% of patients scoring 11-48 and 3.6% of patients scoring ≥49. Compared to these findings, the participants in this study had higher SFNSL scores and a greater proportion of patients classified as having “probable/highly probable SFN and SFN”. These results reflect the characteristics of the study population.

Based on the PROMs results, this study population showed a higher proportion of patients with persistent symptoms and more severe cases compared with previous studies that included outpatients with a broad range of disease severity. These findings likely reflect the characteristics of the study population, as members of the patients’ association tend to represent individuals with systemic symptoms and more advanced disease.

A nationwide survey in Japan [4] reported low prevalence of fatigue (6.6%) and pain (1.5-4.1%). These surveys were not conducted using PROMs but were based on physician-documented data. Consequently, symptoms that patients do not explicitly report may be underestimated. In particular, subjective symptoms, such as fatigue and pain, are prone to discrepancies between physician assessments and patients’ self-reported experiences. Given this background, fatigue and symptoms associated with SFN in sarcoidosis may not be adequately recognized, leading to delays in appropriate management and a consequent decline in HRQoL.

The KSQ GHS score correlated with both the FAS and SFNSL scores (Figure 5), suggesting a relationship between HRQoL and organ-nonspecific systemic symptoms, such as fatigue, and those associated with SFN. Previous studies have also shown a correlation between the KSQ and FAS scores [23,26], indicating that the KSQ captures the influence of fatigue on HRQoL.

Furthermore, we compared the FAS and SFNSL scores between patients with and without OCS use and analyzed the proportion of patients that exceeded the respective cut-off values. In the FAS score, a significantly higher proportion of patients in the OCS group had scores ≥35. Similarly, in the SFNSL score, a significantly higher proportion of patients in the OCS group had scores ≥11.

Because this study was cross-sectional, it did not establish a causal relationship between OCS use and symptoms such as fatigue or those associated with SFN. Several studies [6,11,27] have investigated the relationship between OCS use and HRQoL. Given these findings, appropriate monitoring and management of systemic symptoms, including fatigue and symptoms associated with SFN, are particularly important in patients receiving OCS treatment. Patients receiving OCS may have more severe disease, which could partly account for the higher prevalence of systemic symptoms observed. PROMs such as the KSQ, FAS, and SFNSL provide a valuable means of capturing the subjective burden experienced by patients and support patient-centered clinical management to improve QOL.

Limitations

This study has some limitations. First, the participants were recruited from a patients’ association, which may have led to selection bias, as patients with more severe symptoms, greater difficulties in daily life, and a higher level of interest in their disease were likely overrepresented. Consequently, our findings may not fully represent all patients with pulmonary sarcoidosis nationwide. Additionally, many participants had a long disease duration, which may have contributed to lower HRQoL due to the cumulative impact of chronic symptoms and complications over time. Some participants had comorbidities, such as cancer, which could potentially influence PROs. This possible confounding was not adjusted for KSQ evaluation and should be considered when interpreting the results.

Second, as this study relied on self-reported data, there is the possibility of bias in the reported use of medications and other treatment-related factors.

Nevertheless, PROMs directly reflect patient experiences, and this study highlights the disease burden in a relatively vulnerable population. These findings underscore the importance of providing appropriate care for patients requiring treatment. Future studies should focus on diverse and comprehensive patient populations.

## Conclusions

In Japan, the burden of sarcoidosis has rarely been assessed using PROMs. This study revealed the burden of patients with pulmonary sarcoidosis through a survey using KSQ, FAS, and SFNSL. The median KSQ GHS score was 54.3, indicating reduced HRQoL, with 72.3% of patients experiencing fatigue and 79.7% suspected of having SFN. Furthermore, patients receiving higher daily doses of OCS had significantly lower KSQ Medication scores, and patients on OCS had significantly higher FAS and SFNSL scores, indicating higher treatment burden, fatigue, and symptoms associated with SFN.

These findings highlight the importance of appropriate monitoring and management of systemic symptoms, including fatigue and symptoms associated with SFN, in patients receiving OCS. Furthermore, this study provides important real-world data from Japan and underscores the value of incorporating PROMs into routine care to comprehensively assess HRQoL and the multifaceted burden of sarcoidosis, thereby supporting more patient-centered approaches to treatment aimed at improving patients’ QOL.

## Figures and Tables

**Figure 6:**
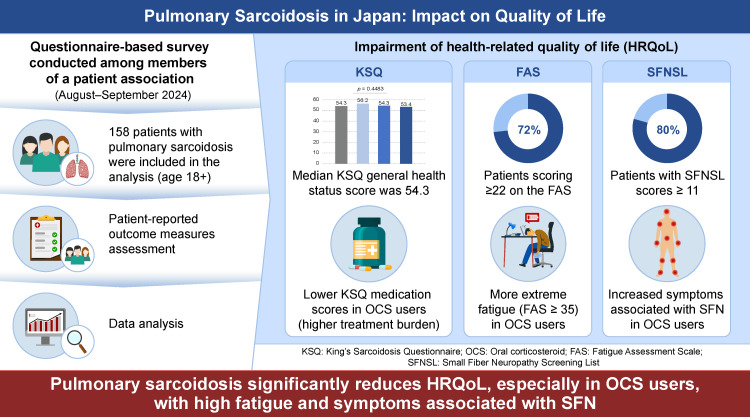
Graphical abstract. KSQ: King’s Sarcoidosis Questionnaire; FAS: Fatigue Assessment Scale; SFNSL: Small Fiber Neuropathy Screening List; OCS: oral corticosteroid; SFN: small fiber neuropathy Image credits: Editage (https://www.editage.com)

## Appendices

Appendix A 

**Table 6:** Distribution of diagnosed patients by organ involvement and gender.

		Overall N = 158	Male n = 23	Female n = 135
					Lesion site, n (%)				
Currently diagnosed				
Lung		126 (79.7)	21 (91.3)	105 (77.8)
Eye		82 (51.9)	6 (26.1)	76 (56.3)
Skin		42 (26.6)	1 (4.3)	41 (30.4)
Heart		52 (32.9)	6 (26.1)	46 (34.1)
Current subjective symptom				
Lung		71 (44.9)	14 (60.9)	57 (42.2)
Eye		79 (50.0)	6 (26.1)	73 (54.1)
Skin		41 (25.9)	1 (4.3)	40 (29.6)
Heart		43 (27.2)	4 (17.4)	39 (28.9)
Previously diagnosed				
Lung		55 (34.8)	4 (17.4)	51 (37.8)
Eye		40 (25.3)	6 (26.1)	34 (25.2)
Skin		40 (25.3)	6 (26.1)	34 (25.2)
Heart		21 (13.3)	4 (17.4)	17 (12.6)

Appendix B 

**Table 7:** KSQ sub-scores by organ involvement. KSQ: King’s Sarcoidosis Questionnaire; GHS: general health status; IQR: interquartile range

Variable	Overall			Lung			Eye			Skin			Heart		
	n	Median	IQR	n	Median	IQR	n	Median	IQR	n	Median	IQR	n	Median	IQR
GHS	157	54.3	18.6	125.0	54.3	18.6	81.0	54.3	16.6	125.0	54.3	18.6	52.0	53.4	20.2
Lung	155	55.1	13.8	123.0	53.8	13.8	81.0	55.1	12.7	123.0	53.8	13.8	51.0	53.8	13.5
Medication	143	80.9	33.1	113.0	80.9	33.1	73.0	80.9	33.1	113.0	80.9	33.1	49.0	66.9	24.4
Skin	146	86.1	46.2	116.0	86.1	46.2	78.0	86.1	46.2	116.0	86.1	46.2	51.0	73.7	46.2
Eye	154	50.6	21.4	122.0	50.6	21.4	81.0	47.2	18.5	122.0	50.6	21.4	51.0	50.6	17.6
GHS lung	154	58.4	11.2	122.0	58.1	10.6	80.0	58.4	10.2	122.0	58.1	10.6	51.0	57.5	11.7
GHS skin	145	53.2	12.9	115.0	53.2	12.9	77.0	53.2	10.5	115.0	53.2	12.9	51.0	51.2	10.8
GHS eye	153	53.8	12.3	121.0	53.8	13.2	80.0	52.5	11.1	121.0	53.8	13.2	51.0	52.9	12.2
GHS lung/skin	144	48.6	10.1	114.0	48.6	10.0	77.0	48.6	7.8	114.0	48.6	10.0	51.0	47.7	9.3
GHS lung/medication	115	60.6	14.8	94.0	60.6	14.7	61.0	60.6	16.0	94.0	60.6	14.7	42.0	59.4	15.1
GHS skin/medication	138	57.5	10.9	109.0	57.5	11.6	72.0	57.5	8.3	109.0	57.5	11.6	49.0	56.0	8.6
GHS lung/skin/medication	137	53.4	9.3	108.0	53.1	8.7	72.0	53.4	7.5	108.0	53.1	8.7	49.0	52.7	9.5

Appendix C 
